# EFSPI/PSI working group on data sharing: accessing and working with pharmaceutical clinical trial patient level datasets – a primer for academic researchers

**DOI:** 10.1186/s12874-016-0171-x

**Published:** 2016-07-08

**Authors:** Rebecca Sudlow, Janice Branson, Tim Friede, David Morgan, Caroline Whately-Smith

**Affiliations:** Roche Products Limited, Welwyn Garden City, UK; Novartis Pharma AG, Basel, Switzerland; University Medical Center, Goettingen, Germany; Ipsen Biopharm (now Pharmaceutical Medicine Group, King’s College, London), Slough, UK; Whately-Smith Limited, Kings Langley, UK

**Keywords:** Data sharing, Data transparency, Patient level data, Clinical trial data

## Abstract

**Background:**

Access to patient level datasets from clinical trial sponsors continues to be an important topic for the Pharmaceutical Industry as well as academic institutions and researchers. How to make access to patient level data actually happen raises many questions from the perspective of the researcher.

**Methods:**

Patient level data access models of all major pharmaceutical companies were surveyed and recommendations made to guide academic researchers in the most efficient way through the process of requesting and accessing patient level data.

**Results:**

The key considerations for researchers covered here are finding information; writing a research proposal to request data access; the review process; how data are shared; and the expectations of the data holder. A lot of clinical trial information is available on public registries and so these are great sources of information. Depending on the research proposal the required information may be available in Clinical Study Reports and therefore patient level data may not need to be requested. Many data sharing systems have an electronic form or template but in cases where these are not available the proposal needs to be created as a stand-alone document outlining the purpose, statistical analysis plan, identifying the studies for which data are required, the research team members involved, any conflicts of interest and the funding for the research.

There are three main review processes - namely having an internal review board, external review board selected by the data holder or an external review board selected by a third party. Data can be shared through Open access i.e. on a public website, direct sharing between the data holder and the researcher, controlled access or the data holder identifies a contract organization to access the data and perform the analyses on behalf of the researcher. The data that are shared will have accompanying documentation to assist the researcher in understanding the original clinical trial and data collection methods. The data holder will require a legally binding data sharing agreement to be set up with the researcher. Additionally the data holder may be available to provide some support to the researcher if questions arise.

**Conclusion:**

Whilst the benefits and value of patient level data sharing have yet to be fully realised, we hope that the information outlined in this article will encourage researchers to consider accessing and re-using clinical trial data to support their research questions.

## Introduction

This article is one of a series of articles developed by the EFSPI/PSI Data Sharing Working Group. The working group consists of medical research statisticians from both pharmaceutical industry and academia. It was set up with the intention of providing knowledge and insights regarding the practical challenges and opportunities of accessing clinical trial data for re-analysis or secondary research purposes.

**Fig. 1:**
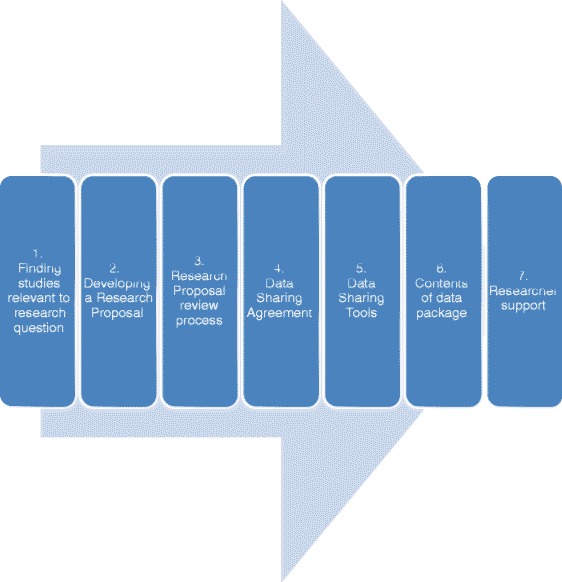
Key steps in the request and access of patient level data. Each step is referenced and described in more detail in the text

The intended audience for this article comprises academic researchers who would like to access patient level data from industry sponsored clinical trials. The article provides a perspective of the activities that happen “behind the scenes” to enable such data sharing to be achieved as well as informing the researcher as to what to expect along the data request process. Figure 1 outlines the seven steps involved in requesting and accessing patient level datasets. This article outlines each of the 7 steps in turn.

The access and the value of re-use of data from clinical trials has been the topic of much debate in recent years [1, 2]. This public and media debate culminated in the European Medicines Agency (EMA) publishing a draft policy on data transparency in 2013 [3], the development of the EFPIA/PhRMA principles [4] for pharmaceutical industry bodies in the Europe and the USA and subsequently the EMA Policy on clinical data for medicinal products for human use in October 2014 [5]. The EMA Policy came into effect on the 1st January 2015. The policy does not include any commitments regarding access to patient level datasets via the EMA, or regulatory documents filed before this date. Many clinical trial funders and sponsors have committed to sharing clinical trial documentation for drugs that are approved after the 1st January 2014 and in many cases also patient level datasets under certain conditions. These conditions include steps to respect patient confidentiality and minimise the risks of re-identification of trial participants. This approach is now widely accepted and supported by statistical communities [6].

The European and American industry bodies representing research-based pharmaceutical companies published the EFPIA/PhRMA principles in 2013 stating that Biopharmaceutical companies are committed to enhancing public health through responsible sharing of clinical trial data (see Table 1 for details). In tandem, many pharmaceutical companies have published their Data Sharing policies. Some have implemented the EFPIA/PhRMA principles as stated, whilst other companies have chosen to broaden the scope of clinical trial data to be shared [7].

**Table 1:** Summary of the EFPIA/PhRMA Principles for Responsible Clinical Trial Data Sharing

1. Enhancing data sharing with researchers	On request from qualified medical and scientific researchers, companies will provide protocols, reports and patient-level clinical trial data for medicines that have been approved in both the EU and US.
	Each company will establish a scientific review board that will include scientists and/or healthcare professionals who are not employees of the company.
	Access will be consistent with patient informed consent and safeguarding privacy.
2. Enhancing public access to clinical study information	Companies will make available synopses of CSRs submitted to US and European regulatory authorities from 1 Jan 2014.
3. Sharing results with patients who participate in clinical trials	Companies will work with regulators to adopt mechanisms for providing a factual summary of clinical trial results and make the summaries available to research participants.
4. Certifying procedures for sharing clinical trial information	Companies will certify on a publicly available web site that they have established policies and procedures to implement these data sharing commitments.
5. Reaffirming commitments to publish clinical trial results	Results from all phase 3 clinical trials and any clinical trial results of significant medical importance should be submitted for publication, whether positive or negative, including results from discontinued development programs.

Patient Data sharing in the era of data transparency is relatively new and processes and tools will continue to evolve. The Institute of Medicine (IoM) [8] the Wellcome Trust [9] the Multi-Regional Clinical Trials (MRCT) unit at Harvard [10] and Tudur Smith et al. on behalf of the Medical Research Council (MRC) Network of Hubs for Trials Methodology [11] all address the challenges and potential principles for future wide-scale patient level data sharing. The objective of this article is to provide practical information to researchers regarding identifying datasets to be requested, the dataset request process, expectations and roles of the study sponsor and expectations of the researcher.

This article will focus on the patient level dataset sharing commitments of clinical trial sponsors (i.e. the electronic datasets created during the course of a clinical trial) and the available methods for third party access to these datasets. It will also give some guidance as to how data requesters should seek access. Access to clinical trial information as summarised within Clinical Study Reports (CSR) will not be addressed in this article. However, we note that Clinical Study Reports provide richer information than that provided in a journal publication. Table 2 provides an outline of the end to end process for requesting access to patient level datasets. The following sections of this Discussion document will address each of these steps in turn.

**Table 2:** Typical End to End Process for requesting Patient Level Data (PLD)

Step	Considerations
1. Develop PLD research proposal	What studies have been conducted?
	Who is the Data Holder?
	Do they have a data access policy and is this study available?
	Could access to the CSR help inform the research proposal development?
	Does the Data Holder have a specific template for the research proposal?
2. Submission and review of research proposal	Who will review it? Independent review panel or within the Data Holder organisation?
	Data Holder’s expectations for sharing data
	Researcher’s expectations for accessing data
3. Dataset preparation for external sharing	How will the data be shared? (open, secure system or other?)
	Data Sharing Agreement review and sign-off
	Data de-identification principles
4. Analysis	Data package contents
	Opportunities to ask questions
5. Reporting / Publication	Data Holder’s expectations regarding publication of the results.
	Referencing the data source
	Any commitments to share the results/manuscript prior to publication?

### Terminology and definitions

For the purposes of this article we will use the following definitions for the terms “Data”, “Data Holder”, “Researcher” and “Anonymised/De-identified” data.

“Data” has many meanings in the context of clinical trial reporting. It can be used to describe publications, clinical study reports, summary statistics and patient level data. Within this article all references to “data” will mean electronic patient level datasets (PLD). Where other data types are mentioned in the text, they will be referred to with an accompanying description.

“Data Holder” will be used to define any organisation that conducts clinical trials and has the rights to share the database of the electronic patient level data for that study. This could include pharmaceutical companies, biotech companies, medical device companies, academic groups and medical charities. We do not consider Contract Research Organisations (CROs) that are paid to conduct studies, collect and analyse clinical trial data on behalf of others to be Data Holders.

“Researcher” will be used to define any individual or group who seeks access to patient level data in order to address a specific research question. Researchers are external to the Data Holder’s organisation and could be academics employed in the public or private sector.

“Anonymised/de-identified” data – Guidance on implementation of the EMA policy 0070 [12] defines anonymisation as “the process of rendering data into a form which does not identify individuals and where identification is not likely to take place”, and anonymised/de-identified data as “data in a form that does not identify individuals and where identification through its combination with other data is not likely to take place”.

The Health Insurance Portability and Accountability Act (HIPAA) [13] defines “de-identified protected health information’ as ‘Health information that does not identify an individual….there is no reasonable basis to believe that the information can be used to identify an individual….”.

Note that terms ‘de-identification’ and ‘anonymisation’ are often used interchangeably in different contexts in the literature.

“Anonymisation/De-identification” of data is the process by which the Data Holder will edit the patient level data to reduce the risk of patient re-identification. This can be achieved through removing, grouping or amending certain data fields (e.g. remove date of birth) and grouping patients (e.g. >89 years of age.

A “Clinical Study Report (CSR)” is the term used in industry for the report generated from a clinical trial. It contains details of the protocol design, a comprehensive overview of the analysis and results and interpretation and conclusions drawn from the data. The CSR is the document that is used to communicate the outcome of the trial to health authorities. A typical CSR (including all appendices) can be in excess of 1000 pages.

## Getting started

The development of a research proposal requesting access to patient level datasets is not without effort on the Researcher’s part. Time will be needed by the Data Holder to review the feasibility of the request, facilitate the scientific review of the proposal and de-identify the datasets and prepare the accompanying documentation. Prior to embarking on the process of writing a research proposal for datasets and submitting it to the Data Holder, the Researcher should consider whether the information within the CSR for the trial would be sufficient to answer the research question of interest. For example, when conducting a meta-analysis using summary data (rather than individual patient data) access to the CSR may be sufficient. Clinical Study Reports contain a richness of information about a clinical trial – including secondary, exploratory and sensitivity analyses that will not have been included within a clinicaltrials.gov posting or a journal article [14]. Clinical Study Reports can be obtained in some cases via requests directly to the sponsor company or through the EMA freedom of information policy. The EMA route should only be used after all other efforts at collaboration have been exhausted and would only be relevant to studies that have been included as part of an European Union (EU) regulatory review.

Accessing a copy of the CSR may be quicker than gaining access to the patient level datasets. If the CSR does not contain the information needed, it will still help inform the Researcher of many more details regarding the trial and may assist in the development of a more complete research proposal for patient level data. If a number of studies (either from one Data Holder or more than one) are of interest, the CSRs will be a useful indication regarding how compatible these trials are likely to be for dataset pooling.

### Step 1: How can a researcher find out which studies have been conducted that fit the research question?

This article assumes that the researcher is familiar with performing literature searches and has identified a set of clinical trials that match their search criteria (e.g. a specific drug, class of drugs or medical condition). Overviews and discussions regarding performing robust literature reviews have been published and the Cochrane Collaboration’s manual is a valuable source of advice [15].

Most clinical trials nowadays are registered on public registries prior to the first patient being recruited and many will have summary results posted within 1 year of the study report being completed. These registries include clinicaltrials.gov [16] for, at minimum, trials with centres in the USA or a drug that was manufactured in the USA. For trials run in the EU, the EUdraCT registry [17] will be a source of useful summary information. The World Health Organisation (WHO) has the International Clinical Trials Registry Platform (CTRP) [18]. However results postings on registries are still not 100 % complete [19] and the issue of clinical trial “discoverability” continues to be a challenge [20].

The unique NCT identifier generated for each study posted on clinicaltrials.gov is also a very useful piece of information to identify a study as some journals require this to be quoted in articles relating to the study. The study sponsor will have a link between the NCT number and their in-house clinical trial naming convention.

The drug approval labels for products can be a useful source of information regarding the studies that were conducted in order for the product to be granted a licence. Both the FDA and EMA have detailed websites [21, 22] where a researcher can view and download these documents. One limitation of the drug label is that the study unique identifier is inconsistently included in these documents. Some drug labels substitute “Study 1”, “Study 2” and “Study 3” (for example) as the names for the pivotal studies. In these situations, a researcher will need to try and link the study characteristics (objectives, patient numbers per arm) with a disclosures database posting to deduce the study identifier. For example in the US registry clinicaltrials.gov, there is a field for “other study ID number” and this may also be informative in identifying the study of interest.

Another area for confusion is the naming of studies. All trials that are run by pharmaceutical companies will have a unique, internal study identifier. This identifier is usually made up of a series of letters and numbers (every company has their own approach to this). It is usually this identifier that is used internally to store all the information regarding a study and so is the most effective name that a researcher can use when interacting with an organisation. Sometimes studies are also given a name (e.g. TENDER). However the name can be used inconsistently to tag information and company databases do not always index on the name. Most articles written about industry sponsored trials rarely include the unique internal identifier, so this will add to the challenge of dataset identification. We recommend that the researcher includes as much information as possible to help facilitate identifying the relevant study data.

#### How to find out which organisation is the data holder for a trial?

The public registry posting for a trial contains details of the “sponsor” – this is a likely to be the Data Holder for the trial and should be a good place to identify who should be approached for access to the study data.

There are also situations where companies have transferred products to other companies, so the original study sponsor as listed on the registries is no longer the Data Holder. Examples of these types of changes are recent divestment of GSK’s oncology products to Novartis [23]. In such situations we recommend that the new licence holder is contacted.

Once the Data Holder has been identified, the researcher will need to find out if the study is available for data access. Access will generally only be available for studies that have completed recruitment and the primary analysis has been reported or the trial was terminated. Studies still recruiting will be out of scope.

#### Which studies are in scope for data sharing?

Data Holders will often provide information regarding access to clinical trial documentation and data via their company websites. These can generally be found by searching “data transparency” or “data sharing” on their site. Articles have been published summarising the data sharing policies of pharmaceutical and biotech companies [7, 24], but in the rapidly changing data sharing landscape these will soon become out of date.

There is no cross-pharma standard regarding the sharing of data from historical studies and the phases of clinical trials to be included. Sponsor commitments can range from all phases (phase 1 through to the post marketing studies) to only the phase 3 pivotal studies. Some sponsors have limited their dataset access to drugs approved since 1st January 2014 whilst others are willing to provide data from earlier filings and for terminated products that never made it through to regulatory approval. The reasons for these differences are varied; they could be based on the text used within the informed consent process historically, whether the metadata for an historic database is easily accessible and whether the creation of a usable data navigation package for the researcher is feasible. Data, programs and dataset documentation generated for trials that were conducted many years ago may be held on old, archived servers and analysis software updates may mean that the analysis code is not easily executable. These factors may mean that the process of dataset anonymization and dataset sharing is difficult for some older studies.

Generally companies have a pre-requisite that the drug and indication must be approved in both the US and EU and that a certain amount of time has passed since the CSR was completed before data is available for sharing with researchers. This time lag is to enable the principal investigators who ran the study sufficient time to publish their work on the study. Some companies require that the results have been published in a journal before any patient level data sharing is possible whilst others base this around a time period (e.g. 18 months after the CSR was completed) [8]. For older studies the decision to grant access to data and the decision to publish may have been handed over to an external Trial Steering Committee (TSC). In these situations the Data Holder will not be able to share data without the Steering Committee’s approval. In the survey conducted by Conroy et al. [25] 42 % of TSCs had a role in determining additional publications to the main report and data sharing.

Some sponsors will provide lists of trials that are available for patient level data requests (e.g. clinicalstudydatarequest.com (CSDR.com) [26], Pfizer [27], Yale University Open Data Access (YODA) [28] whilst others will also provide a route for researchers to request the availability of a specific study (ies) e.g. CSDR.com and YODA.

### Step 2: Best practices for writing a patient level data request research proposal

Many patient level data request systems have an electronic proposal form on their website that the researcher needs to complete as part of the request process. Table 3 provides an outline of the types of information that may be required. This outline may be a useful starter for when there is no structured application form to fill in. Tudur Smith et al. [11] and CSDR.com [26] provide detailed examples of research request documentation.

**Table 3:** Key components of a research request form

Proposal component	Additional notes
Name and affiliation of the lead researcher	
Statement of the Scientific Goals of the Research	
Synopsis of Research Proposal	Lay version may also be needed
Statistical Analysis Plan (SAP)	Including endpoints to be evaluated, analytic methods to used and methods to control for bias in post-hoc or data driven analyses. Should also state whether specific populations are to be analysed e.g. effects of treatment in special patient groups
Studies for which data is requested	Use unique study ID if known and database version required (if the study has been conducted over a long period of time and has been analysed at different follow-up time points)
	Include all studies to be combined including those obtained from other sources (e.g. studies completed by your own institution)
Name and affiliation of other members of the research team	There should be a professionally qualified statistician or confirmation that the proposed research team has the relevant statistical expertise to perform and take responsibility for all statistical analyses should be provided.
	Some Data Holders require CVs or other information as reference.
Conflicts of interest	Both real and potential
Source of Funding	This is the funding source for the researcher.
	Currently Data Holders do not require payment for the preparation and access to the patient level datasets.

The researcher should check if their institution or country requires ethics approval for their proposed work. It is the responsibility of the researcher to get ethics approval and some Data Holders may ask for proof of this as part of their review process.

The researcher should clearly identify the study (or studies) required, preferably using the company unique identifier. Some long running studies (e.g. in oncology and other chronic disease settings like rheumatoid arthritis) may have been analysed and reported for a number of outcome measures and a number of timepoints and as such multiple versions of the database and CSRs will be on file. The request should include the database version required as part of the proposal. If there is a specific publication that was used to identify the study as being required, include the publication detail. These details will help the Data Holder’s data sharing support team to identify the most appropriate dataset for you.

Not every item of “data” that has been collected within the clinical trial will be available to share. Most companies will provide copies of the electronic database for the study, but may not be able to share copies of the completed case report form pages, imaging data (e.g. x-ray films or computed tomography (CT) scans) or genetic data. Studies that are small (less than 50 patients) or have been performed within a rare disease setting may not be available for sharing if it is felt that in these settings, the potential risks of patient identification are too high. These requests will likely be considered on a case by case basis.

A lay summary outlining the key intentions of the research proposal is often required. For the CSDR.com website [26] the lay summary of the research intent is posted for public view whilst the analysis is actively on-going. There are references [29–31] that provide advice regarding writing lay text in the context of medical research.

### Step 3: Who decides whether your research proposal is valid? The role of a review panel

The assessment of a research proposal requesting access to patient level data has two aspects: is the study in question considered to be available for sharing (the Data Holder’s decision) and does the research proposal have sufficient scientific merit (the Review Panel’s decision). The Review Panel can be structured in a variety of ways.

At one extreme, Data Holders can choose to oversee the review themselves by utilising employees of their organisation to do this. Another approach is that they use clinical trial experts outside their organisation to perform this role. The three common types of Review Panel are:

1. **Internal Review Panel.** All members are direct employees of the Data Holder organisation. The advantage of this approach is that the reviewers will have a deep understanding of the product and clinical trial(s) identified and whether the research questions identified have been previously addressed internally (though may not have been published). The downside of this review approach is that the board could be perceived as biased in approving only research that is in keeping with the company’s research interests.
2. **External Review Panel (selected by the Data Holder).** Devolving approval decisions to a group of experts outside the Data Holder’s immediate organisation mitigates some of the concern for biased decision making. However the fact that the experts have been selected by the Data Holder and will be paid directly by the Data Holder can lead to criticism of their true independence.
3. **External Review Panel (selected by a third party).** Using an independent third party (such as the YODA and Janssen collaboration [28] or the Wellcome Trust and clinicalstudydatarequest.com collaboration [26]) for the selection and organisation of the Review Board is the ideal for truly independent decision making. In this model the Data Holder would contract the third party to run the Review Board and the third party would contract experts of their choice to perform the review. The Review Board members would not receive any payments for their work from the Data Holder directly but be paid for their time via the third party.

Some Data Holders (e.g. Amgen [32], Pfizer [33]) have opted for a hybrid approach whereby only those research proposals that have been rejected by the internal review board will subsequently be sent to an external panel for a final assessment (mixture of approach 1 and 2 above).

The Review Board should be made up of a mixture of disciplines. Ideal membership will include physicians and at least one statistician and/or epidemiologist. There may also be representation from medical ethicists, patient advocates and disease area specialists. The Chair of the Review Board will generally be the final decision maker.

Depending on the remit of the Review Board, the review of the Research Proposal may vary from a high level assessment of the research objectives and statistical considerations to a detailed critique of the research in the context of the current research thinking on that specific topic. Some companies make public the metrics for the number of requests received and the number of Review Board approvals and rejections [26, 28].

### Step 4: Data holders’ expectations of researchers

Within the current data sharing systems Data Holders cover the costs of generating the anonymized datasets, collating the data package and paying the licences for the controlled access systems that are used to provide secure access to the data (if applicable). In return there are a number of conditions that the researcher may be required to commit to before being granted access to the data. This may be encapsulated within a legally binding document called a Data Sharing Agreement (DSA) or Data Use Agreement (DUA). As this is a legal document the researcher may need to liaise with the Legal Department at their research institution prior to signing. Many of the data access request portals provide open access to a copy of the DSA text for anyone to view and download. The DSA step is frequently the slowest step to gaining access to the data particularly in cases where text changes are requested, as such it is recommended that researchers contact their legal representatives early in the process (e.g. at submission of the research proposal) for advance warning and advice. The legal review can take from a few days to many months if legal negotiations are requested by the researcher.

The Data Sharing Agreement can include commitments to:

- have an appropriately qualified research team
- ensure that the privacy and confidentiality of clinical trial subjects will be safeguarded with no attempt to establish their identities
- publicly post the Research Proposal and Analysis plan (ideally prior to the analysis being performed). Some data sharing platforms provide this facility. For systems where the Research Proposal plan is not posted by the Data Holder, the Researcher could post on their own academic institution's website.
- only use the data to address the objectives outlined in the Research Proposal and state that it will not be used for any other purpose or shared with any third party.
- rapidly communicate any safety or efficacy issues to regulators and Data Holder(s) if identified. For example, if inconsistencies are found with the original analysis.
- publish results in a peer reviewed journal or other public forum and provide a copy of the publication to the Data Holder. Note some sponsors may request the opportunity to provide a courtesy review prior to publication, but any comments or changes suggested by the sponsor would not be binding.
- attempt to complete research within a fixed time period

Additionally, although not specifically covered in the DSA, good analysis and programming practices (as outlined in ICH E9 [34] and ENCePP Guide on Methodological Standards in Pharmacoepidemiology [35]) should be used.

#### Data holder’s commitments to researchers

Data Holders that have an internal review process for data sharing requests should monitor the progress of the review of research proposals submissions and seek to minimise delays. Following the approval of a data sharing request, the Data Holder should seek to generate the datasets to be shared and accompanying documentation in a reasonable period of time. The progress of generating the materials to be shared should be monitored to minimise delays. Data Holders may have a central team to manage all data sharing activities or individual project teams may have to deliver on individual data sharing responsibilities. Irrespective of the framework being used, Data Holders should ensure that the key stakeholders within the organisation are aware of and trained in the process to ensure full understanding of roles and researcher’s expectations.

### Step 5: How datasets are shared with researchers

There are a number of data access models that could be considered by an organisation. Examples include:

**Open access:** Data is prospectively posted on to a public website. Individual researchers can access these data without having to go through a formal research request review process and they are able to download datasets and hold copies on their own computers. An example is the Immune Tolerance Network (ITN) TrialShare initiative [36].

**Direct Sharing:** Following approval of a data sharing request, the Data Holder provides copies of de-identified data directly to the Researcher. The Researcher contacts the Data Holder directly and they agree to enable the research. Datasets and documentation are sent directly to the researcher (using secure file transfer protocol (SFTP) or other secure electronic transfer system) so the researcher is able to analyse the data on their own local computer systems. An example is the Alzheimer’s Disease Neuroimaging Initiative (ADNI) [37].

**Controlled access:** Following review and agreement of the research proposal by a review panel, data are uploaded by the Data Holder into a secure website along with supporting documentation. The researcher is given access to the secure website and they perform all their programming and analysis within the system. The researcher is able to download analysis results but is not able to download the datasets. There may be the facility for the researcher to upload data and programs into the secure website. Again a data sharing agreement may be set up between the researcher and the Data Holder. Examples are YODA [28] and CSDR.com [26].

Many participants in clinical trials support the concept of making data broadly available to maximize its scientific value for future patients and this interest is driving today’s data sharing principles. It is however the Data Holder’s responsibility to maintain the security of clinical trial subject’s personal information. Access models like controlled access or third party analysis are preferred by the pharmaceutical industry, primarily as they can best ensure patient data confidentiality [8]. Table 4 outlines these options and their pros and cons from both the Researcher’s and Data Holder’s point of view. Open access provides the most flexibilty for Researchers but results in a high risk to patient confidentiality or results in extensively anonymized data that will impact on data useability. The other solutions outlined (direct sharing, controlled access and third party analysis) require increasing levels of complexity and time in order to gain access to the data but will retain dataset useability as the scale of necessary data anonymization will be less.

**Table 4:** Overview of the different patient level data access models and their pros and cons from the Researcher’s and Data Holder’s perspectives

Pros	Cons
OPEN access:	
Researchers: Immediate access for researchers. No pre-requisites needed regarding qualifications or documentation of the research objectives and analysis plan. Data Holder: No need for a research request and review process	Researchers: No guarantee of direct access to the Data Holder if they encounter difficulties in navigating the data or if they have questions regarding the study conduct.
	No knowledge of who else is accessing the data. Potential for overlapping or repeated research questions arising from the same dataset leading to increased chances of errors or increased type 1 errors.
	Data Holder: High risk to patient confidentiality as the data could be combined with other datasets.
	No traceability regarding who has accessed the data, whether they are qualified in statistical analysis and how they have consequently used the data.
	As pre-specification of analysis is not needed nor monitored, there is a risk for data dredging and over-interpretation of findings.
	High internal costs if all trials are required to be anonymized and posted prospectively some of which may never be accessed.
	Resource-intensive.
Direct Sharing	
Researcher: No limitation on the statistical software that can be used.	Researcher: Responsible for the security of any information held on their systems.
Easier to merge and combine data from a variety of sources.	Potential impact on research credibility if collaboration by Researcher is seen as not truly independent from the Data Holder.
Increased opportunity to address data and analysis questions with the Data Holders’ study personnel.	
Data Holder: Potential for identifying synergies where the research is in keeping with research interests of the organization. Potential opportunity to collaborate with the Researcher and address any questions they have during their research work.	Data Holder: Security of the datasets is reliant on the security of the requesters systems. Reliant on the Researcher adhering to the terms of the DSA relating to not sharing data outside the research group and only using the data for research activities that have been approved. Without a DSA there is a risk of data being misused. Level of interaction between research and Data Holder project team could impact on internal resources and timelines for other activities Medium resource-intensive
Controlled Access	
Researcher: Ability to access data from multiple Data Holders in a defined process	Researcher: Required to use only the analysis software supplied within the secure website. May not be the software that they usually use, or with which they are familiar leading to inconvenience and increased chance of erroneous analysis.
Data Holder: Datasets are supplied in a secure environment so the risk of patient identification via merging with other datasets is reduced The named researchers are the only ones who can interact with the data. Ability to compare pre-specified analyses versus published findings. Able to share data with a wide range of researchers with less impact on internal resourcing (compared to Open and Direct Sharing approaches)	Commitments to publish/share their results with the Data Holder may be required. Concern that the work held on the system could be viewed by the Data Holder. Long term access may be tricky requiring archiving procedures. May not be able to use data made accessible within a controlled environment with data provided directly due to different Data Holder sharing strategies. Cannot be combined with controlled access data from other sources. Data Holder: Data are used for purposes beyond that outlined in the original access request (controlled by data sharing agreement). Cost to the sponsor for the website. Relatively low resource intensity
Third Party Analysis	
Researcher: Researcher does not require statistical analysis expertise in the team as this will be provided by the third party.	Researcher: Lack of direct interaction with the data could be frustrating. Reliant on a positive collaboration with the third party analysts.
Able to focus his/her time on interpreting the analyses rather than in data manipulation and programming.	Analysis work may be convoluted as there will need to be a lot of interaction between the third party and the researcher.
Data Holder: The analyses performed on the data is in keeping with the proposal provided by the researcher. Reduced potential for unplanned, additional data explorations.	Data Holder: Cost implications. Not practical for small organisations. Increased chance of mis-understanding of data structures and thus possible quality issues if the Data Holder is not involved.
	Analysis not considered to be independent as Data Holder owns the contract with the third party.
	Relatively low resource intensity but cost could be HIGH

#### Example patient level data models for some pharmaceutical industry data holders

Since the publication of the EFPIA/PhRMA principles (July 2013), many pharmaceutical Data Holder organisations have published their data transparency policies online. The EFPIA Clinical Trial Data Portal [7] is a quick way to find information about a specific pharmaceutical company and what they are willing to share and how to request data. However, as companies continue to publish and update their policies, the individual company website will have the most accurate information regarding the scope of their policy.

Pharmaceutical Data Holders have adopted a variety of approaches to the mechanics of data requests. A number of companies ask that you contact them directly, examples are AstraZeneca [38], Amgen [32], Merck [39], Shire [40]) and Pfizer (via their INSPIIRE portal [33]). Janssen and Bristol-Myers Squibb (BMS) have opted to collaborate with an academic group – Janssen with Yale University (called the YODA project [28]) and BMS with Duke University [41]. In this model the academic group is contracted to act as the gatekeeper for access to clinical trial data by managing the request process, review process and providing information and datasets to the researcher (via secure access system) and liaison with the researcher. Janssen and BMS staff provide the data packages required by the researcher to the Yale and Duke teams respectively, but are not actively involved in any decision making or communicating with the researcher directly.

GSK launched a website for requesting access to GSK trials (studies initiated on/after 2000) in May 2013. Since then a number of companies have collaborated with them to develop the clinicalstudydatarequest.com website. This is a website which enables both single Data Holder and multiple Data Holder requests to be submitted. For example, if a Researcher requires access to clinical trial data from GSK and Roche then they submit one research proposal for all the studies identified, it is reviewed by one Review Board, one Research Agreement is signed and the data will be uploaded by each company into the same area of the controlled access website. Since August 2015 the Wellcome Trust has been responsible for the set-up and management of the independent review component of the process [26].

#### Potential challenges of working with data from multiple sources

The potential insights gained from access to data from multiple Data Holders and from both failed and successful studies will be of great benefit to medical understanding. This vision is not fully realised as yet as not all trial sponsors have policies in place for access to patient level data that includes failed trials or old trials.

Even if the trials that are needed are available the challenges the researcher will face when combining data from different sources should not be underestimated. Most studies will have been set up with few intentions of combining across Data Holders, the case report form questions, drug dictionaries, adverse event dictionaries as well as the database structure itself will all need to be assessed for feasibility of combining. The process of merging data from different sources can take longer than expected and a realistic view of the resource and time needed should be factored into the Researcher’s plans.

In the future, the use of the Clinical Data Interchange Standards Consortium (CDISC) [42] data structure principles may go a long way to mitigate these problems within pharmaceutical industry sponsored studies, but fundamental differences in how questions have been asked or subsequently coded on the case report forms (CRF) will continue to be an issue unless CRF and corresponding meta-data are made publicly available or shared between sponsors and trialists [43].

## Data package contents and researcher support

### Typical dataset structure for pharmaceutical studies

The standard statistical software package that is used in the pharmaceutical industry is SAS (Statistical Analysis System), and as such the datasets from a clinical study will tend to be in a SAS format. This is not the case for non-Pharma sponsors of clinical trials who may use a variety of different analysis software tools. When submitting a new application or supplemental filing to the FDA, the sponsor company is required to submit the electronic patient level data for the studies in the submission such that the FDA Statistical Reviewer can re-analyse the study and re-confirm key conclusions drawn by the company. Standardization of dataset structures for all data domains (e.g. demographic data, laboratory data and adverse event data) and for specific disease endpoints is the goal of the CDISC group [42]. However we are still a long way from one consistent approach across all organisations.

A “typical” clinical trial consists of a number of patient visits and at each visit a number of medical assessments will be made and recorded. This results in databases that can hold thousands of datapoints for just one individual subject. The standard approach is to create “raw” datasets that reflect the data as it was recorded on the case report form and “analysis ready” datasets where all the data derivations are generated. It is the “analysis ready” datasets that are then used as the input to any statistical analyses and data summaries. The principles applied to get from the raw data to the derived datapoints in an analysis dataset will have been outlined in the Statistical Analysis Plan (SAP) and Dataset Specifications. A “typical” phase 3 study can have between 15 and 20 datasets (available as both raw and analysis versions) and can be between 5 and 16 gigabytes in total.

In order to anonymise data, all data will be subject to some editing (datapoints removed) and new patient identifiers (compared to those used in the trial) will be created in order to reduce the chance that an individual will be identified from their clinical trial data. A number of publications have discussed the issue of patient de-identification [44–46] and we will not discuss it any further here. The EFSPI/PSI Working Group article on protecting patient confidentiality will cover best practices on this topic [47].

### Step 6: Contents of a typical data package

Irrespective of the mode of data sharing (open, direct or controlled), a researcher should expect to receive documentation to explain the contents of the datasets provided. Table 5 outlines the minimum information that Data Holders should share with Researchers when access to data is approved. The term “Data Package” is used to describe all the information being shared and is made up of both data and documents to help orientate the Researcher around the datafiles. The documents should include the study protocol, statistical analysis plan and study report as well as more technical documentation. Provision of SAS code and SASlogs are optional and less likely to be included as part of the standard content. Table 5 provides more detail about each of these documents.

**Table 5:** Possible Data Package Contents

Item	Further details
Anonymized Raw datasets	Dataset content reflects the information as recorded on the case report form. These are usually split into a number of raw datasets reflecting the different types of data that have been collected, e.g. adverse events, laboratory assessments, disease specific measurements. Only the datasets required for the research may be provided by some Data Holders
Anonymized Analysis-ready datasets	These datasets will have been derived from the raw datasets and will reflect the additional programming that needs to be applied for the data to be analysis ready. This could be the synthesis of different datapoints to create a single efficacy assessments (e.g. ACR score in RA or a time to disease progression) and could also include derivations and assumptions as a result of missing data. They will also identify the original analysis populations (e.g. ITT, Per Protocol) that were defined in the Statistical Analysis Plan. Researchers should understand the differences between these populations so they can be used appropriately.
Protocol (including any amendments)	The protocol describes the clinical study design, assessment schedule and planned statistical analysis in detail. Small amounts of text may be subject to redaction if they are considered to be commercially confidential.
Annotated Case Report Form	This document provides the link between the data points that were recorded by the investigator onto the paper or electronic case report form and the variable name and dataset location where they are held within the database. This is a key document to help the researcher navigate the database.
Statistical Analysis Plan	This document is written by a statistician prior to the study data being available for analysis. It is a comprehensive outline of the statistical endpoints to be derived and analysis methodology to be used. The 1 to 2 pages of statistical detail from the protocol are expanded into a document that can be 10–20 pages in length.
Dataset specifications	This (alongside the Statistical Analysis Plan) will provide a map of the dataset structure and data variable locations
Clinical Study Report	The CSR will be subject to some redactions in order to preserve patients’ anonymity and in some cases to protect commercially confidential information. The patient level data listings will not be included.
Optional: SAS Programs	In situations where the analysis ready datasets cannot be found, sponsors may choose to share the SAS programs that were used to create the derived datasets and analysis results. Note that copies of SAS programs may not be executable on other systems without some editing.
	In certain cases the SAS code outlining the statistical models used may be shared in order to help the researcher navigate the data and original modelling approach.
Optional: SAS Logs	Limited value as the original SAS program may not be executable on other computer systems or outside of the Data Holder’s standard SAS macro calls.

The IoM report calls for the sharing of the SAS code that was used to generate the datasets and the analysis results. Most companies do not currently share the SAS code that was used as this will not be executable easily on other SAS systems due to references to in-house macros and other SAS standards. However details of the model/analysis statements used to generate the analysis could be shared as they provide the researcher with a good understanding of the model fitted (e.g. random effects, interaction terms, and estimate statements).

### Step 7: Researcher support

It is acknowledged that researchers may need support and advice when first navigating through the complex dataset structures and materials.

It is important to discuss the potential for further support from the Data Holders as the Researchers embark on their work. Table 6 outlines the different scenarios where discussion between the researcher and the Data Holder may be needed or requested. This covers the various stages in the research process when communication between the Researcher and Data Holder may be required, the types of questions and how they may be implemented.

**Table 6:** Different scenarios for communication between Data Holder and Researcher

Communication between data holder and researcher	Purpose	How to implement / alternatives	Consequences if not possible
While researcher is putting together the research proposal	Researcher fully understanding which data have been collected, study design etc.	Possibility for Researchers to raise questions directly regarding data collected on the data sharing company sites.	Higher number of research proposals needing to be rejected or resubmitted following initial review.
During research to clarify understanding of study’s SAP, dataset specifications	Enable the Researcher to understand data and the analysis already conducted.	Ensure complete documentation is provided by data holder to eliminate this as much as possible	Lack of knowledge of data collected potentially leading to inappropriate analysis.
Sharing the completed analysis, interpretation and proposed publications	Sponsor is aware prior to publication of any difference in interpretation of results	Sponsor requests to be informed up front of publication. The alternative is to take a risk and deal with receiving information in parallel to it being in public domain	Differing results based on analyses of anonymized data could lead to different interpretation and raise either justifiable or unnecessary concerns in scientific and public domains

Most Data Holders will offer a support/question route for the researcher. However this is likely to be limited to questions about the data and will not be for more general statistical analysis advice (e.g. How do I fit a certain model in SAS?) or to provide clinical support.

For those companies sharing data via a controlled access system such as the SAS Clinical Data Transparency Tool ®, support will be provided for navigation and set up by the provider of the tool.

## Conclusions

This article addresses the key steps in requesting access to patient level data and offers advice on how to navigate through the request process. The key challenge that remains for all researchers is discoverability of the trials that have been conducted by both pharma and academia; until there is 100 % compliance with the commitments around registry postings this will continue to be an issue [19, 20].

Different data sharing models and platforms mean that true cross product data collation is still not a reality. However as most platforms (e.g. YODA, CSDR.com) and companies (e.g. Pfizer) are all using the SAS Clinical Trial Data Transparency Tool as the means to securely share the datasets and documents, cross platform sharing could be feasible in the future. Researchers are encouraged to make enquiries to trial sponsors in situations where the studies required are available through different platforms in order to see if a workaround is possible.

The new era of access to patient level data is still in its infancy and Data Holders and researchers continue to learn from their experiences so far. Enhanced data access holds the promise to increased scientific knowledge and understanding whilst balancing the potential risks to patient identification. At this time the benefits and challenges of patient level data sharing have not been fully realised and we look forward to seeing how this new world of transparency develops.

## Summary

The objective of this article is to provide a primer for academic researchers when accessing and working with clinical trial data from other organisations. The main points of consideration which are highlighted are for the researcher to first find out what information is needed and whether indeed patient level data is required. Once the need for patient level data is established a recommendation is given as to what a research proposal should contain. The Researcher needs to understand the Research Proposal review process, access commitments and data access mechanism offered by the Data Holder.. Finally the expectations of the data holder are discussed in terms of data sharing agreements and also what support can be made available to the researchers with whom their data is being shared.

## Abbreviations

ACR, American college of rheumatology; ADNI, alzheimer’s disease neuroimaging initiative; BMS, Bristol-Myers Squibb; CDISC, clinical data interchange standards consortium; CRF, case report form; CRO, contract research organisation; CSDR.com, clinicalstudydatarequest.com; CSR, clinical study report; CT scan, computer tomography scans; CTRP, clinical trials regsitry platform; DSA, data sharing agreement; DUA, data use agreement; EFPIA, European federation of pharmaceutical industries and associations; EFSPI, European federation of statisticians in the pharmaceutical industry; EMA, European medicines agency; ENCePP, European network of centres for pharmacoepidemiology and pharmacovigilance; EU, European union; EudraCT, European clinical trials database; FDA, food and drug administration; GSK, glaxosmithkline; ICH, international conference on harmonisation; IoM, institute of medicine; ITN, immune tolerance network; ITT, intention to treat; MRC, medical research council; MRCT, multi regional clinical trials center of Harvard and Brigham and women’s hospital; NCT, number used as identifier in clinicaltrials.gov; PhRMA, pharmaceutical research and manufacturers of America; PLD, patient level data; PSI, statisticians in the pharmaceutical industry; RA, rheumatoid arthritis; SAP, statistical analysis plan; SAS, statistical analysis system; SFTP, secure file transfer protocol; TSC, trial steering committee; US, United States of America; WHO, world health organisation; YODA, yale university open data access.

## References

[1] AllTrials Campaign. [http://www.alltrials.net]. Accessed 11 April 2016.

[2] British Medical Journal Open Data Campaign. [http://www.bmj.com/open-data]. Accessed 11 April 2016.

[3] European Medicines Agency. European Medicines Agency Draft policy on publication and access to clinical-trial data. 24 June 2013 [http://www.ema.europa.eu/docs/en_GB/document_library/Other/2013/06/WC500144730.pdf] Accessed 11 April 2016.

[4] European Federation of Pharmaceutical Industries and Associations (EFPIA) and Pharmaceutical Research and Manufacturers of America (PhRMA). Principles for responsible clinical trial data sharing: our commitment to patients and researchers. 18 July 2013. [http://www.phrma.org/sites/default/files/pdf/PhRMAPrinciplesForResponsibleClinicalTrialDataSharing.pdf]. Accessed 11 April 2016.

[5] European Medicines Agency. European Medicines Agency policy on publication of clinical data for medicinal products for human use. 2 Oct 2014. [http://www.ema.europa.eu/docs/en_GB/document_library/Other/2014/10/WC500174796.pdf]. Accessed 11 April 2016.

[6] Fletcher C, Driessen S, Burger HU, Gerlinger C, Biesheuvel E (2013). European Federation of Statisticians in the Pharmaceutical Industry's position on access to clinical trial data. Pharm Stat.

[7] European Federation of Pharmaceutical Industries and Associations. Clinical Trial Data Portal Gateway [http://transparency.efpia.eu/responsible-data-sharing/efpia-clinical-trial-data-portal-gateway]. Accessed 13 April 2016.

[8] Institute of Medicine (2015). Sharing clinical trial data: maximizing benefits, minimizing risk.

[9] Varnai P, Rentel MC, Simmonds P, Sharp TA, Mostert B, de Jongh T. Assessing the research potential of access to Clinical Trial Data. Report to Wellcome Trust 2015 [http://www.wellcome.ac.uk/stellent/groups/corporatesite/@msh_peda/documents/web_document/WTP058912.pdf]. Accessed 13 April 2016.

[10] MRCT Center of Harvard and Brigham and Women’s Hospital. Proceedings of Promoting Clinical Trial Data Transparency Conference. March 2015 [http://mrctcenter.org/wp-content/uploads/2015/11/2015-04-30_mrct_data_transparency_conf_proceedings.pdf]. Accessed 13 April 2016.

[11] Tudur Smith C, Hopkins C, Sydes M, Woolfall K, Clarke M, Murray G, Williamson P. Good Practice Principles for Sharing Individual Participant Data from Publicly Funded Clinical Trials. Version 1, Apr 2015. [http://www.methodologyhubs.mrc.ac.uk/files/7114/3682/3831/Datasharingguidance2015.pdf] Accessed 13 April 2016. 10.1186/s12916-015-0532-z PMC4682216 26675031

[12] European Medicines Agency. External guidance on the implementation of the European Medicines Agency policy on the publication of clinical data for medicinal products for human use. 2 March 2016. [http://www.ema.europa.eu/docs/en_GB/document_library/Regulatory_and_procedural_guideline/2016/03/WC500202621.pdf] Accessed 11 March 2016.

[13] US Office for Civil Rights (OCR). Guidance Regarding Methods for De-identification of Protected Health Information in Accordance with the Health Insurance Portability and Accountability Act (HIPAA) Privacy Rule. 26 Nov 2012. [http://www.hhs.gov/sites/default/files/ocr/privacy/hipaa/understanding/coveredentities/De-identification/hhs_deid_guidance.pdf] Accessed 11 March 2016.

[14] Doshi P, Jefferson T (2013). Clinical study reports of randomised controlled trials: an exploratory review of previously confidential industry reports. BMJ Open.

[15] Higgins JPT, Green S. (editors). Cochrane Handbook for Systematic Reviews of Interventions Version 5.1.0. The Cochrane Collaboration, 2011. [http://handbook.cochrane.org/]. Accessed 13 April 2016. [http://www.ncbi.nlm.nih.gov/pmc/articles/PMC3196245/]

[16] ClinicalTrials.gov [https://clinicaltrials.gov/] Accessed 13 April 2016.

[17] EUdraCT public website [https://eudract.ema.europa.eu/index.html] Accessed 13 April 2016.

[18] WHO International Clinical Trials Registry Platform [http://www.who.int/ictrp/en/] Accessed 13 April 2016.

[19] Anderson M, Chiswell K, Peterson E, Tasneem A, Topping M, Califf R (2015). Compliance with Results Reporting at ClinicalTrials.gov. N Engl J Med.

[20] Miller JE, Korn D, Ross JS (2015). Clinical trial registration, reporting, publication and FDAAA compliance: a cross-sectional analysis and ranking of new drugs approved by the FDA in 2012. BMJ Open.

[21] FDA Approved Drug Products [http://www.accessdata.fda.gov/scripts/cder/drugsatfda/] Accessed 13 April 2016.

[22] European Medicines Agency. European Public Assessment Reports. [http://www.ema.europa.eu/ema/index.jsp?curl=pages/medicines/landing/epar_search.jsp&mid=WC0b01ac058001d124]. Accessed 11 April 2016.

[23] GSK Press Release 22 April 2014 [http://us.gsk.com/en-us/media/press-releases/2014/gsk-announces-major-3-part-transaction-with-novartis-to-drive-sustainable-sales-growth-improve-long-term-earnings-and-deliver-increasing-returns-to-shareholders/]. Accessed 13 April 2016.

[24] Krumholz H, Gross C, Blount K, Ritchie J, Hodshon B, Lehman R, Ross J (2014). Sea change in open science and data sharing: leadership by industry. Circ Cardiovasc Qual Outcomes.

[25] Conroy E, Harman N, Lane J, Lewis S, Murray G, Norrie J, Sydes M, Gamble C (2015). Trial Steering Committees in randomised controlled trials: A survey of registered clinical trials units to establish current practice and experiences. Clin Trials.

[26] Clinical Study Data Request.com. [https://www.clinicalstudydatarequest.com/]. Accessed 13 April 2016.

[27] Pfizer. Data Access Requests. [http://www.pfizer.com/research/clinical_trials/trial_data_and_results/data_requests]. Accessed 13 April 2016.

[28] The Yale University Open Data Access (YODA) Project. [http://yoda.yale.edu/]. Accessed 13 April 2016.

[29] Cochrane Collaboration. Standards for the reporting of Plain Language Summaries in new Cochrane Intervention Reviews (PLEACS) Version 3.0 [http://editorial-unit.cochrane.org/sites/editorial-unit.cochrane.org/files/uploads/PLEACS_0.pdf]. Accessed 13 April 2016.

[30] MRCT Center of Harvard and Brigham and Women’s Hospital. Return of aggregate results. [http://mrctcenter.org/return-results]. Accessed 13 April 2016.

[31] British Library. Writing about biomedical and health research in plain English: A guide for authors. [http://www.access2understanding.org/wp-content/uploads/2014/11/Access-to-Understanding-writing-guidance_v1.pdf] Accessed 11 April 2016.

[32] Amgen. Clinical Trial Transparency, Data Sharing and Disclosure Practices. [http://www.amgen.com/about/data_sharing.html]. Accessed 15 April 2016.

[33] Pfizer. Transparency of clinical trial data. [http://www.pfizer.co.uk/content/transparency-clinical-trial-data] Accessed 15 April 2016.

[34] ICH E9 Expert Working Group. Statistical principles for clinical trials: ICH harmonized tripartite guideline. Stat Med. 1999;18(15):1905–42. [doi: 10.1002/(SICI)1097-0258(19990815)18:15%3C1903::AID-SIM188%3E3.0.CO;2-F] [http://www.ncbi.nlm.nih.gov/pubmed/10532877] 10532877

[35] The European Network of Centres for Pharmacoepidemiology and Pharmacovigilance (ENCePP). Guide on methodological standards in pharmacoepidemiology (Revision 4). EMA/95098/2010. [http://www.encepp.eu/standards_and_guidances/documents/ENCePPGuideofMethStandardsinPE_Rev4.pdf] Accessed 15 April 2016.

[36] Immune Tolerance Network. TrialShare. [http://www.immunetolerance.org/node/1121]. Accessed 15 April 2016.

[37] Alzheimer’s Disease Neuroimaging Initiative (ADNI). Sharing Alzheimer's research data with the world. [http://adni.loni.usc.edu/] Accessed 15 April 2016.

[38] AstraZeneca Group of Companies. Data request portal. [https://astrazenecagroup-dt.pharmacm.com/DT/Home]. Accessed 15 April 2016.

[39] Merck. Company procedure on access to clinical trial data. [http://www.merck.com/clinical-trials/pdf/Procedure_on_access_to_clinical_trial_data_2016.pdf]. Accessed 15 April 2016.

[40] Shire. Our commitment to transparency. [http://www.shiretrials.com/en/our-commitment-to-transparency]. Accessed 15 April 2016.

[41] Bristol-Myers Squibb. Disclosure commitment. [http://bms.com/clinical_trials/pages/disclosure.aspx]. Accessed 15 April 2016.

[42] Clinical Data Interchange Standards Consortium. CDISC mission and principles. [http://www.cdisc.org/CDISC-Vision-and-Mission]. Accessed 15 April 2016.

[43] Dugas M, Jöckel K-H, Friede T, Gefeller O, Kieser M, Marschollek M, Ammenwerth E, Röhrig R, Knaup-Gregori P, Prokosch H-U. Memorandum “Open Metadata”: Open access to documentation forms and item catalogs in healthcare. Methods Inform Med. 54: 376–8. doi: 10.3414/ME15-05-0007. [http://www.ncbi.nlm.nih.gov/pubmed/26108979]. 10.3414/ME15-05-0007 26108979

[44] Hrynaszkiewicz I, Norton M, Vickers A, Altman D (2010). Preparing raw clinical data for publication: guidance for journal editors, authors, and peer reviewers. BMJ.

[45] Hughes S, Wells K, McSorley P, Freeman A (2014). Preparing individual patient data for clinical trials for sharing: the GlaxoSmithKline approach. Pharm Stat.

[46] TransCelerate Biopharma Inc. Data de-identification and anonymization of individual patient data in clinical studies – a model approach. [http://www.transceleratebiopharmainc.com/wp-content/uploads/2015/04/Data-Anonymization-Paper-FINAL-5.18.15.pdf] Accessed 16 April 2016.

[47] Tucker K, Branson J, Dilleen M, Hollis S, Loughlin P, Dixon MJ, Williams Z. Protecting patient privacy when sharing patient-level data from clinical trials. BMC Med Res Methodol. 2016 (accepted for publication). 10.1186/s12874-016-0169-4 PMC4943495 27410040

